# Emerging and Adjunctive Therapies for Spinal Cord Injury Following Acute Canine Intervertebral Disc Herniation

**DOI:** 10.3389/fvets.2020.579933

**Published:** 2020-10-15

**Authors:** Melissa J. Lewis, Nicolas Granger, Nick D. Jeffery, The Canine Spinal Cord Injury Consortium (CANSORT-SCI), Sarah A. Moore

**Affiliations:** Author Affiliations: DACVIM-Neurology, Associate Professor, Neurology and Neurosurgery, Department of Veterinary Clinical Sciences, The Ohio State University College of Veterinary Medicine, Columbus, OH, United States; DACVIM-Neurology, Professor of Neurology/Neurosurgery; Distinguished Chair of Gerontology, Department of Clinical Sciences, North Carolina State University College of Veterinary Medicine, Raleigh, NC, United States; DACVIM-Neurology, Professor; Chair, and Head, Department of Small Animal Clinical Sciences, College of Veterinary Medicine and Biomedical Sciences, Texas A&M University, College Station, TX, United States; DAVCIM-Neurology, Assistant Professor of Neurology, Department of Veterinary Clinical Sciences, Purdue University College of Veterinary Medicine, West Lafayette, IN, United States; Professor Neurology & Neurosurgery; Professor in Small Animal Clinical Sciences, College of veterinary Medicine, Texas A&M University, College Station, TX, United States; Professor and Service Head, Neurology and Neurosurgery, Department of Veterinary Clinical Sciences, College of Veterinary Medicine, The Ohio State University, Columbus, OH, United States; Department of Clinical Sciences, Colorado State University, Fort Collins, CO, United States; Department of Clinical Science and Services, Royal Veterinary College, Hawkshead Lane, Hatfield, Hertfordshire, United Kingdom; The Royal Veterinary College, University of London, Hawkshead Lane, Hatfield, United Kingdom and CVS Referrals, Bristol Veterinary Specialists at Highcroft, Bristol, United Kingdom; Faculty of Veterinary Medicine, Institute of Veterinary Pathology, Leipzig University, Leipzig, Germany; Division of Clinical Neurology, Department for Clinical Veterinary Medicine, Vetsuisse Faculty, University of Bern, Bern, Switzerland; Department Small Animal Medicine and Surgery, University of Veterinary Medicine Hannover, Hannover, Germany/Europe; Neurology and Neurosurgery, Department of Veterinary Medicine and Surgery, University of Missouri, Columbia, MO, United States; Department of Small Animal Medicine and Surgery, University of Veterinary Medicine Hannover, Hannover, Germany/Europe; ^1^Department of Veterinary Clinical Sciences, Purdue University College of Veterinary Medicine, West Lafayette, IN, United States; ^2^The Royal Veterinary College, University of London, Hertfordshire, United Kingdom; ^3^CVS Referrals, Bristol Veterinary Specialists at Highcroft, Bristol, United Kingdom; ^4^Department of Small Animal Clinical Sciences, Texas A & M College of Veterinary Medicine and Biomedical Sciences, College Station, TX, United States

**Keywords:** alternative therapies, interventions, dog, intervertebral disc disease, cell transplantation, spinal cord injury, canine

## Abstract

Some dogs do not make a full recovery following medical or surgical management of acute canine intervertebral disc herniation (IVDH), highlighting the limits of currently available treatment options. The multitude of difficulties in treating severe spinal cord injury are well-recognized, and they have spurred intense laboratory research, resulting in a broad range of strategies that might have value in treating spinal cord-injured dogs. These include interventions that aim to directly repair the spinal cord lesion, promote axonal sparing or regeneration, mitigate secondary injury through neuroprotective mechanisms, or facilitate functional compensation. Despite initial promise in experimental models, many of these techniques have failed or shown mild efficacy in clinical trials in humans and dogs, although high quality evidence is lacking for many of these interventions. However, the continued introduction of new options to the veterinary clinic remains important for expanding our understanding of the mechanisms of injury and repair and for development of novel and combined strategies for severely affected dogs. This review outlines adjunctive or emerging therapies that have been proposed as treatment options for dogs with acute IVDH, including discussion of local or lesion-based approaches as well as systemically applied treatments in both acute and subacute-to-chronic settings. These interventions include low-level laser therapy, electromagnetic fields or oscillating electrical fields, adjunctive surgical techniques (myelotomy or durotomy), systemically or locally-applied hypothermia, neuroprotective chemicals, physical rehabilitation, hyperbaric oxygen therapy, electroacupuncture, electrical stimulation of the spinal cord or specific peripheral nerves, nerve grafting strategies, 4-aminopyridine, chondroitinase ABC, and cell transplantation.

## Introduction

Current treatment for acute canine intervertebral disc herniation (IVDH) can be divided into medical/conservative or surgical management. The decision as to which to pursue depends largely on the severity of neurologic signs. These might be due to either reversible or irreversible damage to the spinal cord itself, with some resulting from tissue ischemia that is difficult to counteract and others resulting from spinal cord compression that are easily reversible. Medical management commonly consists of activity restriction, pain, and anti-inflammatory medications. The goals are: (i) to avoid further disc herniation to minimize additional damage to the spinal cord; (ii) provide pain relief; (iii) allow the extruded disc material to gradually dissipate by phagocytosis over time; (iv) and leave the ruptured disc annulus to seal by fibrosis over time. Surgical intervention is used to alleviate persistent spinal cord compression. The reader is directed to the article, “Current approaches to the management of acute thoracolumbar disc extrusion in dogs” for more information regarding the evidence for the commonly applied treatment options.

Neither medical nor surgical management currently aim to repair the damaged intervertebral disc, nor heal the injured spinal cord, and there are limits to the recovery that can be attained. Severe injuries still result in incomplete recovery and unsatisfactory functional status. Although largely understandable, with restoration of function to spinal cord injured individuals recognized as a holy grail for centuries, this failure has prompted a vast effort in neuroscience research. The aim is to develop strategies that directly target the injury within the spinal cord, limit the extent of secondary injury, facilitate regeneration of axons, or increase compensatory plasticity of the surviving tissue. Many neuroprotective and neuro-regenerative therapies have shown promise in pre-clinical experimental models but few have made it beyond this phase and when they have, repeatedly failed to successfully translate to humans or dogs with naturally-occurring spinal cord injury (SCI) (1, 2). In fact, it has been reported that only about one-third of animal studies for any disease considered to have a high likelihood of translation into human medicine actually progressed to the clinical trial stage and even fewer were associated with any currently available intervention (3, 4). While this should not deter researchers and clinicians from seeking novel treatment options for the injured spinal cord, it does highlight the huge hurdles facing such work and underscores the difficulty of the problem. Importantly, knowledge of what has been done, successful or otherwise, is crucial to broadening our understanding mechanisms of injury and recovery, developing new techniques, or adapting and combining previously suggested treatment modalities for application to clinical populations.

This review will summarize adjunctive or emerging therapies that have been proposed as treatment options for dogs with acute IVDH. We will focus on data available from companion dogs with naturally-occurring SCI but also include data on experimental dogs where relevant. This is the information that we hope will be most useful to veterinary clinicians and might soon be applicable in the neurology clinic. We will focus on therapies that can be applied following acute SCI (within 1 month of injury) but also discuss therapies that might aid in repairing the spinal cord or restoring function in the subacute to chronic patient (> 1 month from injury). We have divided treatments in those delivered at or close to the injury site and those delivered systemically.

## Adjunctive Therapies Implemented in the Acute Phase

### Local/Lesion-Based Interventions

A variety of locally administered interventions have been proposed in the treatment of SCI that are applied directly to the lesion site in addition to, or *in lieu* of, decompressive surgery. These include laser therapy, application of electromagnetic fields or oscillating electrical fields, adjunctive surgical techniques, locally-applied spinal cord hypothermia, and cell transplantation. Cell transplantation strategies will be discussed in the subacute to chronic section in the second part of this review.

### Laser Therapy

Low-level laser therapy or photobiomodulation has been a reported therapy for various injuries, including SCI. In the nervous system, it has been proposed to enhance neuronal metabolism and sprouting and to decrease glial scar formation and the immune response (5, 6). While not fully understood, the mechanisms of action are reported to include inhibition of NF-kB (which reduces expression of pro-inflammatory mediators) and stimulation of cytochrome oxidase (which might help to optimize oxidative metabolism) (5, 6). In an unblinded, unrandomized prospective study of non-ambulatory paraparetic or paraplegic dogs with IVDH, with or without intact pain perception at enrolment and treated surgically, laser therapy applied post-operatively (for 5 days or until independent ambulation was achieved) was compared to dogs that did not receive additional therapy. The reported time to achieve independent ambulation was shorter in the laser therapy group (3.5 days) compared to untreated control dogs (14 days) (7). However, the characteristics of the laser employed were not detailed making it difficult to try to replicate results. In contrast, a blinded, randomized prospective study evaluating post-operative laser therapy with or without physical rehabilitation in non-ambulatory dogs undergoing surgery for IVDH revealed no difference in recovery (8). Importantly, both studies included a relatively small number of dogs in each treatment group, including few with severe injury, did not incorporate pre-study sample size calculations, and only looked at short-term outcome variables. No adverse events attributable to laser therapy were reported.

### Electromagnetic and Electrical Field Therapies

While application of a pulsed electromagnetic field (PEMF) device to the site of injury has been most widely studied in pain and wound repair, PEMF therapy has been reported to reduce back and neck pain in people and possibly improve recovery from SCI in an experimental model in cats (9–13). The mechanism of action of PEMF in pain relief is likely multifactorial and there is limited evidence in central nervous system injury that it can aid in promoting axonal regeneration or sparing of surviving axons (9, 10). In a recent blinded, randomized prospective clinical trial of paraplegic dogs with absent pain perception secondary to IVDH that underwent surgery, PEMF reduced post-operative incisional pain (as measured by increased mechanical sensory thresholds) compared to sham-treated control dogs. The authors also reported a possible neurologic benefit based on measuring injury severity using plasma GFAP concentration and recovery of proprioceptive placing (14). However, sample size was small and multiple outcomes were evaluated.

Oscillating electrical field therapy, which is suggested to enhance axonal regrowth and improve functional recovery, has been applied to spinal cord-injured animals (15). In paraplegic deep pain negative dogs secondary to IVDH treated surgically, oscillating electrical field therapy was delivered post-operatively via electrodes sutured to the edges of the laminectomy site and attached to an implantable device. Treatment was administered for a variable number of weeks post-operatively and the device and therapy were well-tolerated. Dogs treated with the electrical fields had improved neurologic outcomes at 6 weeks and 6 months after surgery compared with sham-treated dogs (15, 16). Despite initial promise, logistical, and technical issues hindered further development of this treatment modality.

### Local Hypothermia

Locally-applied spinal cord hypothermia has been rarely reported as a treatment for SCI in dogs in experimental studies (17–20). Hypothermic conditions (4–6°C) were applied to the spinal cord initiated at 15 min to 4 h after injury and maintained for variable durations ranging from 1 to 18 h. In these studies, hypothermia was reported to improve functional outcome in experimentally-injured dogs compared to untreated controls with a possible additive benefit in combination with other therapies. However, reported drawbacks included extensive technical and personnel demands, the potential for inadvertent damage to spinal cord through prolonged hypothermia and lack of information on long-term outcomes or sequelae (17). This technique has not been reported in dogs with naturally-occurring injury secondary to IVDH but mean body temperature was identified as an exploratory variable worthy of further evaluation in prospective studies in dogs with IVDH (21). Local and systemic hypothermia continue to be investigated in human medicine (22, 23).

### Adjunctive Surgical Techniques to Spinal Cord Decompression: Durotomy and Myelotomy

The role and indications for decompressive surgery as well as fenestration as a standalone technique for acute IVDH are outlined in the companion article in this issue, “Current approaches to the management of acute thoracolumbar disc extrusion in dogs.” Adjunctive surgical techniques of durotomy and myelotomy are summarized below.

Durotomy has been investigated as a means to decompress a swollen spinal cord, to improve spinal cord blood flow and oxygen delivery and to evaluate for gross myelomalacia as a prognostic indicator (24–27). Durotomy with or without duroplasty has been reported to have positive effects in multiple experimental rodent and human SCI studies; however, reported functional impact is variable, adverse effects are possible, and controlled studies are lacking (24). In experimental studies in dogs, immediate but not delayed (by 2 h) durotomy was reported to enhance recovery rate and overall neurologic outcome (25, 27, 28). In clinical canine patients, durotomy has typically been reserved for severely affected dogs. Blaser et al. demonstrated that durotomy combined with decompressive hemilaminectomy in dogs with IVDH (of varying severity ranging from ambulatory paraparesis to paraplegia with intact pain perception) transiently increased intraoperative spinal cord blood flow, although it returned to normal or lower within 15 min (26). There was no association detected between durotomy and 1-day post-operative neurologic outcome. However, none of the included dogs were those that have the most to benefit from durotomy (i.e., those paralyzed with absent pain perception at presentation), thereby potentially limiting the generalizability of these results. In an additional retrospective study of 48 paraplegic dogs that were deep pain negative secondary to IVDH, no difference was detected in recovery of ambulation between those that did or did not receive a durotomy in conjunction with hemilaminectomy (29), although confounding by severity is a clear possibility in this study.

More recently, contrasting evidence has been provided by Takahashi et al. who reported on 116 paraplegic deep pain negative dogs with thoracolumbar IVDH treated with hemilaminectomy alone (*n* = 65) or hemilaminectomy *plus* durotomy (*n* = 51) (30). A large proportion of dogs recovered following durotomy vs. hemilaminectomy alone (56.9 vs. 38.5%). The low rate of recovery in the non-durotomy group (compared to most published reports of a 50–60% success rate with decompressive surgery) was attributed to inclusion only of cases that had imaging features associated with poor prognosis. Notably, no dogs in the durotomy group compared to 14 in the hemilaminectomy-only group developed progressive myelomalacia. In another recent report, “extended durotomy” of four vertebral lengths centered over the site of herniation was also investigated in 26 consecutive paraplegic dogs that were deep pain negative secondary to thoracolumbar IVDH (31). Of the 26 dogs included in the study, 4 dogs were lost to follow-up while 16/22 remaining dogs recovered independent ambulation within 6 months (with 15/16 also recovering continence) (31). No adverse events were attributable to the extended durotomy; one dog developed progressive myelomalacia. These studies together reinvigorate the discussion as to whether durotomy might be beneficial in dogs with severe injury, especially in preventing development of progressive myelomalacia. Additional information is needed regarding single vs. extended durotomy, the role of duroplasty, patient selection among severely affected dogs, and the risk and functional impact of long-term consequences such as fibrosis that might negatively impact neurologic function.

Dorsal midline myelotomy has been reported as a treatment for SCI to decrease intramedullary pressure, increase the oxygen interface, remove necrotic debris, and release noxious vasoactive substances trapped in the spinal cord post-injury (24, 32). In an experimental canine model, myelotomy in combination with dimethyl sulfoxide (DMSO) appeared to have an additive benefit on neurologic recovery compared to other experimental treatment combinations, although myelotomy alone was not evaluated (32). In another study on experimental SCI followed by myelotomy, there was immediate improvement in sensory evoked potential amplitude in 2/5 dogs (33), suggesting temporary improvement in conduction, but it is unclear if this is sustained or associated with functional benefit. Myelotomy performed in normal dogs has been associated with extensive gray matter necrosis including destruction of ventral horn motor neurons in some dogs (34). However, clinical impairment from the procedure was generally mild to moderate and improved over several weeks as long as the lumbar intumescence was avoided (34). While a positive effect has been reported in 80% of pre-clinical animal studies, there are no published studies in naturally-occurring injury in dogs and very limited data available in humans (24). The lack of controlled studies is likely attributable to the invasiveness of myelotomy and perceived potential to exacerbate secondary injury and for long-term adverse sequelae.

## Systemic Compound/Medication-Based Therapies

A variety of systemic or “whole dog” interventions have been applied to treat dogs with IVDH. Administration of some type of systemic medication or chemical as a neuroprotective strategy for the treatment of acute IVDH has been reported to be recommended by up to a quarter of specialist veterinarians (35). This varied greatly by treatment type, being highest for steroid administration (34% of boarded surgeons, 23% of boarded neurologists recommended) and <10% for other interventions (35). Adjunctive, non-medication-based therapies typically applied post-operatively were also variably reported as part of an integrated treatment strategy. Physical rehabilitation was most common and recommended by approximately half of treating veterinarians (35).

### Corticosteroids

Corticosteroids are a commonly administered adjunctive therapy for the treatment of IVDH in dogs. Methylprednisolone (MPSS) at so-called “shock doses” has received the most attention and been most extensively examined but dexamethasone has also been investigated in dogs (17–19, 32, 36–43). MPSS has been advocated as a neuroprotective treatment for acute SCI through its mitigation of secondary injury primarily through amelioration of lipid peroxidation, other free radical, and oxidative damage and reperfusion injury (44). Although initial results of human clinical trials appeared supportive of use of high dose MPSS for treatment of SCI, subsequent re-analysis of the data cast doubt on the original treatment effect and highlighted risks of adverse effects (45–47). In dogs with IVDH, a benefit for MPSS has not been identified and complications have been reported (36, 42, 43, 48) and the use of MPSS remains controversial (46). The role of corticosteroids in this population is discussed in depth in the companion article “Current approaches to the management of acute thoracolumbar disc extrusion in dogs.”

### Polyethylene Glycol

Polyethylene glycol (PEG) a hydrophilic polymer capable of fusing cell membranes has been infrequently investigated as a treatment for acute SCI with inconsistent results. In an acute canine spinal cord transection model, immediate application of PEG at the site of injury was determined to be beneficial and to re-establish anatomic continuity (49). In a study of dogs with acute paraplegia with absent pain perception due to IVDH, intravenous PEG administration appeared safe, and associated with modestly improved neurologic status 6–8 weeks after injury and surgery compared to what might be expected in similarly affected dogs not receiving PEG (50). In a more recent clinical trial of acute paraplegic dogs with absent pain perception due to IVDH, no benefit was demonstrated for PEG compared to placebo (36).

### Matrix Metalloproteinases

Matrix metalloproteinases (MMPs) are released by cells to degrade the extracellular matrix. MMPs, specifically MMP-9 and MMP-12, have been shown to be upregulated following SCI and are implicated in the deleterious secondary injury cascade. In two prospective studies, Levine et al. evaluated a broad-spectrum MMP inhibitor, GM6001, in dogs treated surgically for IVDH resulting in acute (<48 h) non-ambulatory paraparesis or paraplegia (51, 52). All dogs were treated immediately before decompressive surgery with the compound, GM6001, combined with DMSO (*n* = 81), DMSO alone (*n* = 84), placebo (*n* = 41), or received no treatment (*n* = 20). Transient injection site reactions were common in the GM6001 treated dogs (which could have compromised the blinding) and a subset (*n* = 6) developed self-limiting musculoskeletal signs, but it was otherwise well-tolerated. Treatment with GM6001 with DMSO resulted in improved neurologic recovery compared to placebo but was not different compared to DMSO alone. While efficacy for treatment with this MMP inhibitor was not demonstrated with regard to sensorimotor recovery, it did increased long-term bladder compliance.

### Dimethyl Sulfoxide

Dimethyl sulfoxide (DMSO) is most often used as a vehicle to improve drug solubility but has been uncommonly investigated as an intervention for brain and SCI (38, 52). Its purported benefit in central nervous system trauma has been attributed to a reduction in edema, diuretic, anti-inflammatory and vasodilatory effects, and cellular protection from mechanical damage (38, 53). In several studies utilizing an experimental weight drop model, dogs were treated with DMSO (1–4.5 g/kg/d in 40% solution with 0.9% NaCl) alone or in combination with other experimental therapies and compared to control dogs (38, 53–55). In most, DMSO was reported to be beneficial when initially administered within 1 h of induced trauma although one study reported no benefit and no clear synergistic effect was observed by combining DMSO with dexamethasone or other experimental therapies. As outlined above, DMSO (1 g/kg) was shown to be beneficial compared to placebo in a clinical trial of dogs with IVDH (52). However, Hoerlein et al. also investigated this in acute spinal cord trauma in dogs and found it not to be useful compared to dexamethasone (56). Toxicity due to DMSO was not observed in any of the reported studies and further investigation is warranted regarding its potential therapeutic effect in this population.

### Other Compounds/Medications

N-acetylcysteine (NAC) is a precursor of glutathione with potent antioxidant as well as anti-inflammatory and neuronal protective properties that has been proposed as a treatment for acute SCI (57). In a cohort of 70 dogs undergoing surgery for acute IVDH, NAC administered IV prior to decompressive surgery showed no benefit compared to placebo with regard to neurologic outcome or rate or recovery (58). There is only anecdotal reference of veterinarians using other antioxidants such as coenzyme Q10 or vitamin E following SCI in dogs (35, 59). While optimizing nutritional status, weight management, and diets to reduce fecal volume in incontinent dogs are variably implemented as part of post-injury management in dogs, there is no evidence to support specific antioxidant nutraceutical supplementation or nutritional strategies to treat dogs with acute SCI.

There are other rarely reported interventions with limited, mostly experimental evidence in dogs. Analogs of the hypothalamic hormone, thyrotropin releasing hormone (TRH), have been reported to inconsistently improve outcomes after SCI in humans and experimental models (59). In a pilot study of dogs with IVDH, a benefit of a TRH analog was not identified compared to no treatment (60). Crocetin, a carotenoid that increases oxygen diffusion in plasma, was investigated in an experimental weight drop model as a means to counteract local hypoxia and subsequent ischemic necrosis following SCI. Results showed improved neurologic function in crocetin-treated dogs at 4 weeks post-injury compared to control dogs (61). Hyperosmotic agents, mannitol, urea and hypertonic dextrose, have also been evaluated with the goal of reducing swelling (32, 53, 54, 62) but did not appear to improve neurologic recovery compared with control dogs. Improvement in spinal sensory evoked potentials did occur following mannitol infusion in one study (32, 53, 54, 62). Phenytoin, an anticonvulsant, was explored as a SCI treatment based on experimental evidence that it decreases edema of neural tissues through inhibition of antidiuretic hormone and inactivation of catecholamines (40). In an experimental dog model, phenytoin resulted in improved outcome compared to untreated dogs and was at least as effective as dexamethasone, although hypotension and respiratory depression were possible adverse effects (40). Neither reserpine, an alkaloid medication used to treat high blood pressure, nor chlorpromazine, a phenothiazine with various psychiatric and other uses, were effective as treatments for experimentally induced injury in dogs (32, 39). There is no convincing evidence for the use of these compounds in dogs with acute SCI due to IVDH.

## Systemic Non-medication-based Therapies

Various systemic, non-medication-based therapies have been advocated for the treatment of acute SCI in dogs including physical rehabilitation, hyperbaric oxygen therapy, and electroacupuncture.

### Physical Rehabilitation

Physical rehabilitation in dogs recovering from surgery due to IVDH is being increasingly utilized, recommended by 58% of board-certified surgeons and neurologists surveyed (35, 63). While timing of initiation and specific protocols vary, physical rehabilitation in the neurologic patient typically consists of some combination of passive range of motion, massage, cold or warm packing, assisted balance, standing, coordination and land treadmill, or over-ground walking exercises and aquatic therapy such as underwater treadmill walking or swimming (64). It can be performed on an in-patient or out-patient basis in dogs with specific aspects tailored to patient function (e.g., underwater treadmill walking sessions are typically initiated once motor function is present). Additional specific therapies including therapeutic laser therapy, acupuncture, and neuromuscular electrical stimulation are variably included (63, 64).

For details on the currently available evidence regarding the role of physical rehabilitation in dogs recovering from IVDH, the reader is referred to the companion article “Current approaches to the management of acute thoracolumbar disc extrusion in dogs” in this issue. While there have been relatively few studies performed in this population and the results have been mixed, early post-operative initiation of rehabilitation has been determined to be safe with no associated adverse events or increased post-operative pain (8, 65–70). Inclusion of dogs of variable neurologic severity limits making direct comparisons between studies and conclusions regarding efficacy. Additionally, the role of physical rehabilitation in medically managed presumptive or confirmed IVDH has not been evaluated. Additional investigation of physical rehabilitation in dogs recovering from IVDH is warranted focusing on optimization of protocols (e.g., specific modalities, timing, and duration) and development of validated, objective outcome measures such as the Finnish neurological function testing battery for dogs (FINFUN) (71).

### Hyperbaric Oxygen Therapy

Hyperbaric oxygen therapy has been uncommonly reported as a treatment for acute SCI. The proposed mechanism is to increase the tissue partial pressure of oxygen and counteract the adverse effects of spinal cord hypoxia associated with injury (72). There is limited experimental evidence in dogs suggesting a potential benefit of hyperbaric oxygen therapy compared to untreated controls but no additive effect was appreciated when combined with DMSO (54, 72). There are no reports in dogs with IVDH though it is used in clinical cases by some veterinarians (35).

### Electroacupuncture

Electroacupuncture has been occasionally reported as a therapy for acute SCI in dogs (73, 74). Its mechanism of action is unknown, but it might have analgesic and anti-inflammatory effects as well as facilitating axonal repair and regrowth (37, 63). In an experimental canine SCI model, electroacupuncture (initiated 48 h post-injury and continued every other day) resulted in improved rate of recovery compared to untreated controls; the benefit appeared synergistic with concurrent MPSS (37). In one retrospective and two prospective case series of dogs with thoracolumbar IVDH of variable severity, electroacupuncture (administered 1–3 ×/week for 1–6 months between studies) was reported to be more effective than decompressive surgery alone for regaining ambulation (75), and was associated with shorter time to walking and a greater proportion of dogs becoming ambulatory compared to medical management alone (73, 74). There was no significant difference in recovery among deep pain negative dogs managed medically with or without electroacupuncture (74). Study limitations for the prospective studies included lack of blinding or randomization, use of historical controls and small sample size within each neurologic grade (74, 75). There is equivocal evidence that electroacupuncture decreases the severity and duration of post-operative pain in dogs with IVDH (74, 76). Electroacupuncture has also been combined with stem cell transplantation in a small group of dogs chronically (> 3 months) deep pain negative following acute IVDH (77). This pilot study showed these interventions were feasible and safe but case numbers in each treatment group were small.

## Adjunctive Therapies Implemented in the Subacute-To-Chronic Phase

Treatment strategies are also being explored for dogs with permanent impairment following acute SCI. These are typically applied in the subacute-to-chronic stage (> 1 month from the time of injury) and include spinal cord radiation, electrical stimulation of the spinal cord or specific nerves below the injury, nerve grafting, 4-aminopyridine, chondroitinase ABC delivery, and cell transplantation.

### Spinal Cord Radiation

Spinal cord radiation has been evaluated in rodents and in an experimental model in Beagles (78). Radiation of the injured cord aims to interfere with the cell cycle to counteract the development of localized chronic inflammation, reduce glial scar formation, and facilitate axonal regrowth and healing (78, 79). In Beagles treated with daily radiation for 2 weeks following spinal cord hemitransection, there was reduced astrocyte and microglial activation, reduced expression of inflammatory mediators, improved long-range axonal regeneration, and improved locomotor recovery (79). This therapy has not been reported in dogs with naturally-occurring injury secondary to IVDH.

### Functional Electrical Stimulation

Functional electrical stimulation has not yet been reported in dogs, but a wearable device is being developed that might be useful in dogs with incomplete recovery from acute IVDH (80). Short-term, low-intensity electrical stimulation of the spinal cord with or without stem cell transplantation has been performed in chronically paraplegic dogs (81). While electromyographic changes in pelvic limb muscles implied improvement in motor conduction, further investigation would be necessary to optimize therapy and determine if there is a clinical benefit (81). Additionally, there is experimental evidence and data in dogs with naturally-occurring injury for electrical stimulation of peripheral nerves or nerve roots to aid in restoration of urination and defecation (82–84). Electrical sacral nerve stimulation in dogs is covered in the companion article “Bladder and bowel management in dogs with spinal cord injury” in this issue.

### Peripheral Nerve Grafting

Grafting of peripheral nerves from above the injury level (e.g., specific intercostal nerves) into the distal portion of experimentally transected spinal cord has also been performed in dogs with the goal of harnessing the regenerative potential of the peripheral nervous system (85–87). Nerve to nerve or nerve root grafting techniques have also been reported as therapies that aim to restore function while bypassing the spinal cord lesion directly. Toreih demonstrated the feasibility of intercostal to gluteal nerves and ilioinguinal and iliohypogastric to femoral nerves in a dog spinal cord hemisection model (88). Six months following these nerve transfer procedures, there was clinical and electrophysiological evidence of some recovery of hip, gluteal and knee function, though spontaneous improvement is well-known to occur in spinal cord hemisection models and could have resulted in the improvement observed. Vagophrenic nerve anastomosis was also shown to be anatomically feasible in dogs with the ultimate goal to provide a conduit for restoration of respiratory function after severe cervical SCI (89). Nerve anastomoses to reinstate bladder function have also been performed experimentally in dogs (90–94).

### Molecular Compounds Given in the Chronic Phase of SCI

Additional compounds that have been investigated in chronically impaired dogs include 4-aminopyridine (4AP) and chondroitinase ABC. 4-aminopyridine is a potassium channel antagonist that has been shown to restore hind limb motor function in some dogs with chronic thoracolumbar SCI (95, 96). This effect is mediated through enhancement of central conduction via anatomically intact axons traversing the site of injury as well as direct synaptic effects (97–99). Response following oral administration is highly variable between individual dogs with a minority regaining independent ambulation (96, 98). Lack of predictable response and narrow therapeutic window have limited widespread use of this medication among chronically paralyzed dogs.

Chondroitinase ABC is an enzyme that degrades chondroitin sulfate proteoglycans which are key components of the glial scar and inhibitors of axonal regeneration following SCI (100). This has led to active research regarding the use and optimization of chondroitinase ABC to treat SCI (100). A prospective clinical trial of intraspinal injection of a long acting chondroitinase ABC in dogs with naturally occurring severe SCI reported functional improvements compared to sham controls including improved thoracic to pelvic limb coordination and three dogs with restoration of ambulation (101). In an experimental canine model, the combination of chondroitinase ABC with mesenchymal stem cell transplantation was also reported to improve neurologic deficits and enhance neural regeneration (102). However, this study had a small number of dogs, lacked blinding of the observations and the locomotor outcome measure might not reflect voluntary movements (102).

## Cell Transplantation Strategies

Transplantation of cells into the spinal cord has been investigated in dogs either after creating an experimental spinal cord lesion or after naturally-occurring SCI. This involves predominantly stem cell therapies such as mesenchymal stem cells of various origin, neural stem cells, or bone marrow-derived mononuclear cells but other fully differentiated cells have been used such as olfactory glial cells, olfactory mucosal cells, Schwann cells, or macrophages. Most commonly, the cell transplants are administered via intraparenchymal or intrathecal injection but intravenous delivery has also been reported.

### Cell Transplantation for Spinal Cord Repair in Experimental Dogs

Placement and inflation of a ventral epidural balloon has been used to produce experimental compression and contusion of the dog spinal cord (103, 104). Developed in the seventies by investigators such as Kobrine and Griffiths, this technique has the advantage of producing a closed injury, without the need to open the spinal canal via a laminectomy, and causes more vascular injury (ischemia) than weight-drop models (105, 106), although lesions lack reproducibility. Using this model, the effect of canine and human umbilical cord blood-derived mesenchymal stem cells, adipose-derived stem cells (some genetically modified), or bone marrow-derived mesenchymal stem cells (107–117) has been tested. These cell transplants were reported to improve locomotor function, but experimental groups consisted of small numbers (between two and five), observers were not blinded and tail support was used when testing locomotion [which is likely to trigger “involuntary” stepping pelvic movements that are independent from brain connections (118, 119)]. However, histopathological data demonstrated survival of some transplanted cells, albeit with limited integration within the host spinal cord, suggesting that the locomotor improvement could have been due to secretion of trophic or growth factors into the region of injury (107).

A canine hemisection model in which a gel seeded with human neural stem cells was placed immediately into the hemisected spinal cord gap (120, 121) showed better locomotor recovery and more ascending sensory axons in dogs receiving cells alone in one study (121) and no effect in another study (122).

A canine transection model has been reported from groups in China recently, testing the effect of collagen-based biomaterial loaded with human umbilical cord-derived mesenchymal stem cells (123), human placenta-derived mesenchymal stem cells (124), or bone marrow-derived mesenchymal stem cells differentiated into neuron-like cells (125). Recipients of cells had improved locomotor scores compared to controls but remained non-ambulatory and the studies were not blinded. Interestingly, cells survived up to 6 months.

A group from Egypt described a compression/contusion model using a “clip” placed on the spinal cord at the L4 spinal cord segment; neural-induced bone marrow derived stem cells were then injected intrathecally by lumbar puncture 2 weeks after the injury by a blinded investigator (126). The injury initially caused paralysis and loss of pain perception in all dogs, but those receiving the cell transplant had much greater recovery of motor function compared to controls. Further, cells could be found surviving in the lesion at 16 weeks after injection. This work represents a lesion and intervention paradigm that are much closer to clinical injuries than other experimental models.

In summary, there is a growing number of transplantation experiments originating primarily from Korea and China which have generally low power and follow the same experimental pattern with varying cell types. One group in Korea translated their experiment to the clinic by transplanting adipose-derived stem cells (112) to 9 companion dogs with paraplegia and no deep pain (127) but trial design lacked clear inclusion criteria and blinding. Therefore, the utility of these treatments for clinical populations remains to be validated in randomized, blinded studies. A cautious approach has been followed by other laboratories, for example, McMahill et al. at the University of California Davis Medical Center, where they have transplanted canine epidermal neural crest stem into normal canine spinal cord (128) and focused on developing strong outcome measures using cell tracking with magnetic resonance imaging and detailed gait analysis. They showed survival of cells at 3 weeks post-transplantation and are likely now envisaging clinical trials in companion dogs with naturally-occurring lesions. There is also uncertainty as to which cell type, stem cells or other differentiated cells, should be prioritized. For example, two other groups have postulated that transplantation of Schwann cells purified from peripheral nerves or nerve roots could be a repair strategy worth pursuing following SCI in dogs (129, 130). Additionally, transplantation strategies can be leveraged to investigate application of biologics (e.g., chondroitinase ABC) to the lesion site as a means to promote cell survival, regrowth, or mitigate inhibition of axonal regeneration.

### Cell Transplantation Within the Spinal Cord in Dogs With Naturally-Occurring Injury

One of the best known cell type studied in dogs is olfactory ensheathing cells, which are not stem cells but fully differentiated cells located within the olfactory mucosa and olfactory bulb, forming an interface between the peripheral and central nervous systems (131–133). Olfactory ensheathing cells have been reliably cultured for a long time in neuroscience (134), including in dogs (135–138) and have been used in clinical applications (139, 140). They are recognized for their regenerative properties when transplanted within a lesion of the central nervous system. In particular, they are able to form channels guiding axonal regrowth (141) and to remyelinate axons (142). In a randomized controlled trial in dogs with irreversible chronic SCI, autologous olfactory ensheathing cells obtained from the nasal mucosa have been shown to improve thoracic to pelvic limb coordination (143). However, these cells were not able to restore brain-controlled functions such as urinary continence, prompting research into strategies to improve their efficacy. More recently, olfactory ensheathing cells have been engineering to express the chondroitinase ABC enzyme that degrades the glial scar (144), though these have not yet been transplanted into dogs.

In the last decade, there has been an increasing number of publications from Japan, Brazil, Turkey, and India testing different cell transplants in dogs with naturally-occurring injury. First in Japan, two groups reported that autologous bone marrow stromal or mononuclear cell transplants were safe in 7 (145) and 1 (146) dogs with chronic paraplegia and absent deep pain.

Since 2014, institutions in Brazil have reported seven trials testing safety or efficacy of various cell transplants (within the spinal cord or in the sub-arachnoid or epidural space) in small series of companion dogs, sometimes with concomitant spinal cord decompression, or in association with other alternative therapies such as electroacupuncture (77) or low-intensity electrical stimulation (81). The cells tested have been either autologous bone marrow mesenchymal stem cells (147), allogenic fetal bone marrow stem cells (148), allogenic canine adipose tissue-derived mesenchymal stem cells (81, 149, 150) or immature dental pulp stem cells (77, 151).

The follow-up duration in these studies was usually of several months. Taken together, the results suggest an improvement of locomotor function, based on an increase in locomotor scores. However, these cases rarely achieved scores suggesting unassisted ambulation and for those that did walk again, they remained deep pain negative suggesting that the locomotion could have been independent of the therapeutic intervention (i.e., “spinal walking”). Interestingly, in some dogs, there was reported recovery of deep pain (147, 148) but no concomitant recovery of locomotion. These findings could indicate that the transplanted cells have a beneficial effect. However, data are limited and these studies also illustrate the heterogeneity of clinical lesions and the need to increase case numbers to better assess the efficacy of cell transplant techniques.

In Turkey, Besalti et al. transplanted intramedullary neurogenically-induced bone marrow-derived mesenchymal stem cells 42 days after the initial injury (152). They conducted detailed follow-up over 12 months and found that 2 out of 13 dogs recovered somatosensory evoked potentials and magnetic motor evoked potentials, while some other dogs had improved gait scores (6/13) and regained deep pain sensation. Bhat et al. in India also reported a trial in 44 dogs testing bone marrow mesenchymal stem cells without decompressive surgery (153). The authors claimed improved deep pain sensation and locomotion, but the change compared to the control group remained clinically small.

Altogether, the results of various cell transplantation studies in dogs are encouraging, although most studies remain of low power and preliminary. They have proven safety, but the recovery is always limited to a proportion of studied dogs and recovery of one function at a time, either locomotion, continence, or pain perception. This perhaps suggests that other factors than the actual treatment led to the change in function and highlights the severity of lesions and difficulties in repairing them. A consensus on which intervention holds the greatest promise would be useful to then apply in large multicenter trials in dogs, where evaluation of efficacy could be investigated with greater power.

## Conclusions

In conclusion, we have outlined a variety of therapeutic strategies that have been applied to dogs with SCI in both the acute as well as subacute-to-chronic settings. These range from those applied to the spinal cord directly to systemic treatments and with variable goals from repair to compensation. While some techniques are more promising than others, they all serve to highlight the challenges in treating severe SCI and in developing successful treatment options for a heterogeneous clinical population. Moving forward, multimodal approaches to therapy building on conventional treatment options will likely be most successful.

## Author Contributions

ML, NG, and NJ participated in manuscript conception, preparation, and editing with the first two (ML and NG) contributing equally. The additional members of the CANSORT-SCI consortium contributed to manuscript conception, editing, and review.

## Conflict of Interest

The authors declare that the research was conducted in the absence of any commercial or financial relationships that could be construed as a potential conflict of interest.
